# The Expression of Adipose Tissue-Derived Cardiotrophin-1 in Humans with Obesity

**DOI:** 10.3390/biology8020024

**Published:** 2019-04-13

**Authors:** Jacqueline Stephens, Eric Ravussin, Ursula White

**Affiliations:** Pennington Biomedical Research Center, Baton Rouge, LA 70808, USA; jsteph1@lsu.edu (J.S.); eric.ravussin@pbrc.edu (E.R.)

**Keywords:** gp130 cytokines, cardiotrophin-1, adipose tissue, abdominal, femoral, obesity, metabolic health, clinical variables

## Abstract

Cardiotrophin-1 (CT-1) is a gp130 cytokine that was previously characterized for its effects on cardiomyocytes and identified as a marker of heart failure. More recent studies reported elevated circulating levels of CT-1 in humans with obesity and metabolic syndrome (MetS). However, a subsequent rodent study implicated CT-1 as a potential therapeutic target for obesity and MetS. Adipose tissue (AT) is broadly acknowledged as an endocrine organ and is a substantial source of CT-1. However, no study has examined the expression of adipose-derived CT-1 in humans. We present the first analysis of CT-1 mRNA expression in subcutaneous AT and its association with clinical variables in 22 women with obesity and 15 men who were 40% overfed for 8-weeks. We observed that CT-1 expression was higher in the subcutaneous abdominal (scABD) than the femoral (scFEM) depot. Importantly, we reveal that scFEM but not scABD, CT-1 expression was negatively associated with visceral adiposity and intrahepatic lipid, while positively correlated with insulin sensitivity in obese women. Also, men with higher CT-1 levels at baseline had less of a decline in insulin sensitivity in response to overfeeding. Our data provide new knowledge on the regulation of adipose-derived CT-1 in obesity and during weight gain in response to overfeeding in humans and suggest that CT-1 may play a protective role in obesity and related disorders.

## 1. Introduction

In recent years, gp130 receptor ligands, including ciliary neurotrophic factor (CNTF) and interleukin (IL)-6, have been shown to modulate energy balance and investigated as potential therapeutic targets for obesity and insulin resistance [1]. Cardiotrophin (CT)-1 is a gp130 cytokine that was first characterized for its hypertrophic and cytoprotective effects on cardiomyocytes [2,3] and elevated levels of CT-1 were subsequently recognized as a marker of cardiovascular disease risk and heart failure [4,5,6,7]. More recent studies support an additional role for CT-1 in the pathogenesis of metabolic disease. Elevated circulating CT-1 levels have been observed in humans with obesity [7,8], metabolic syndrome (MetS) [8] and type 2 diabetes (T2DM) [9], suggesting that CT-1 may play a pathophysiological role in obesity-related complications. Conversely, others have reported lower CT-1 levels in overweight and obese individuals compared to normal-weight [9,10,11]. Furthermore, a compelling rodent study from Moreno-Aliaga et al. revealed CT-1 to be an important and beneficial regulator of glucose and lipid metabolism with potential applications for the treatment of obesity and MetS [12]. Mice lacking CT-1 developed obesity, insulin resistance and dyslipidemia, while chronic administration of CT-1 reduced body weight, improved insulin resistance and resolved hepatic steatosis in obese mice [12,13]. Hence, the findings regarding the functions of CT-1 in human obesity and metabolism are inconsistent. 

Previous studies assessed only circulating plasma levels of CT-1, without considering the tissue source. Adipose tissue (AT) serves as the primary reservoir for energy storage but has also emerged as a key endocrine organ, producing important factors (adipokines) that mediate whole-body metabolism and influence the development of obesity and related disorders. In addition to the heart, skeletal muscle, liver and kidney, AT has been identified as a substantial source of CT-1 relative to other metabolic tissues [8]. Hence, the regulation and secretion patterns of CT-1 in AT likely account for changes in circulating levels and may influence metabolism. Despite the characterization of CT-1 as an adipokine [14], no study has examined the association between adipose-derived CT-1 and metabolic health outcomes in humans. 

In the present analysis, we examined the mRNA expression of CT-1 in subcutaneous AT and its association with clinical variables in a population of 22 healthy women with obesity (30 ± 6 years of age; 32.4 ± 2.5 kg/m^2^), as well as a cohort of 15 lean men who were overfed by 40% more than their baseline energy requirements for 8-weeks (25 ± 4 years of age; BMI 24.7 ± 2.1 kg/m^2^). Studies have shown that the distribution of adipose tissue, rather than overall obesity, may be a stronger predictor of metabolic health risks, as upper-body adiposity confers a higher risk of obesity-related disorders, while lower-body fat may be metabolically protective [15,16,17]. Given these opposing associations, we analyzed both subcutaneous abdominal (scABD) and femoral (scFEM) depots in the women. We observed that CT-1 expression was higher in the scABD than the scFEM depot. Our study demonstrates for the first time that CT-1 levels in the scFEM but not the scABD, were negatively associated with visceral adiposity and intrahepatic lipid, while positively correlated with insulin sensitivity, as assessed by the Matsuda Index and HOMA-IR, in women with obesity. In addition, men with lower CT-1 levels at baseline had a greater decline in glucose disposal rate (i.e. insulin sensitivity) in response to 8-weeks of overfeeding.

## 2. Materials and Methods

### 2.1. Participant Characteristics

#### 2.1.1. Study 1

The experimental design and protocol for this cross-sectional study have been previously described in detail [18]. Healthy women with obesity were recruited based on the following inclusion criteria: 18-40 years of age, body mass index (BMI) 27–38 kg/m^2^, fasting plasma glucose (≤ 110 mg/dL), blood pressure (≤ 140/90 mmHg), absence of major organ disease, normal urinary and blood laboratory tests, weight stability (± 3.2kg) for ≥ 3 months and no self-reported significant changes in diet or physical activity in the previous month. Exclusion criteria included a history of diagnosed diabetes, liver or heart disease; chronic use of medications with metabolic effects; or use of medications or surgical procedures that cause weight gain or loss. Subjects with HIV, hepatitis B or hepatitis C were also excluded, as were pregnant or breastfeeding women. The Institutional Review Board at Pennington Biomedical Research Center approved the protocol and all participants provided written, informed consent. (Clinicaltrials.gov Identifier: NCT01748994).

#### 2.1.2. Study 2

Similarly, the experimental design and protocol for the overfeeding study intervention have been previously described in detail [19]. Healthy men were recruited according to the following inclusion criteria: 20–40 years of age, body mass index (BMI) 22–32 kg/m^2^, normal urinary and blood laboratory tests and weight stability (± 2.5kg) for the previous 6 months. Exclusion criteria included a history of chronic disease (diabetes, heart or liver disease, hypertension, gastrointestinal disorder), eating disorders, a BMI > 32 kg/m^2^ at any point in life, HIV, hepatitis B and hepatitis C. Pennington Biomedical Research Center’s Institutional Review Board approved the protocol and all subjects gave written, informed consent. (Clinicaltrials.gov Identifier: NCT01672632).

Baseline Weight Stabilization and Overfeeding Intervention: Prior to the 56-day (8-week) overfeeding period, participants completed a 2-week measurement of free-living energy expenditure using doubly labeled water (DLW) to determine baseline energy requirements. During the second week of DLW, participants consumed an isocaloric diet (60% carbohydrate, 25% fat, 15% protein). Baseline energy requirements were calculated as the average of the measured 2-week energy expenditure by DLW and the 1-week calorie level provided during feeding at weight maintenance, which was multiplied by 1.4 to determine the overfeeding prescription. This calorie level was initiated on the first day of overfeeding and was maintained until the last day (Day 56).

During the 8-week overfeeding period, all meals were prepared and administered by the Pennington Biomedical Research Center’s Metabolic Kitchen using a 5-day rotating menu and contained 41% carbohydrate, 44% fat and 15% protein. Meal times at breakfast, lunch and dinner were supervised by the dietary staff to ensure that all foods were eaten. Participants were free-living the remainder of the time. On Day 56, subjects were fed a weight maintaining diet for 3 days before metabolic testing.

### 2.2. Study Procedures (Baseline and Post-Overfeeding Intervention)

Anthropometric characteristics (height; metabolic weight; waist-hip ratio (WHR); mean blood pressure (1/3 (systolic BP - diastolic BP) + diastolic BP)) were collected. Fasting plasma total triglycerides (TRIG), high density lipoprotein (HDL), low density lipoprotein (LDL) and total cholesterol (CHOL) were measured.

#### 2.2.1. Body Composition

Dual-energy x-ray absorptiometry (DXA) was performed using the General Electric Lunar iDXA to measure total body fat. Scans were analyzed with the enCORE software version 13.60.033. scABD AT and visceral AT (VAT) volumes were defined and quantified with magnetic resonance imaging (MRI) using a 3.0T scanner (GE, Discovery 750w) by obtaining ~ 581 images from the dome of the liver to the pubic symphysis. Images were analyzed by a single trained analyst. Estimates of scABD AT and VAT volumes were converted to mass using an assumed density of 0.92 kg/L.

Intrahepatic lipid (IHL) was measured by proton magnetic resonance spectroscopy (^1^H-MRS) on a 3.0T whole body imaging and spectroscopy system (GE, Discovery 750w System) using a commercially available ^1^H body coil. IHL content was determined with jMRUI (Java-Based Magnetic Resonance User Interface) with peak areas expressed relative to the peak area of an external oil phantom (peanut oil).

#### 2.2.2. Oral Glucose Tolerance Test

In women with obesity, insulin sensitivity was assessed by the 2-hour oral glucose tolerance test (OGTT) and calculated using the Matsuda Index (10,000/square root of (fasting glucose x fasting insulin) × (mean OGTT glucose x mean OGTT insulin)) [20].

#### 2.2.3. Hyperinsulinemic Euglycemic Clamp

In men, insulin sensitivity was measured using a two-step hyperinsulinemic euglycemic clamp. Insulin was infused for 180 min at 10 mU/min m^2^ and for 150 min at 50 mU/min·m^2^. Glucose (20%) was infused at a variable rate to maintain plasma glucose concentration at 90 mg/dL. The average amount of glucose required during the final 30 min of each insulin infusion step (glucose infusion rate (GIR)) was considered the measure of insulin sensitivity [21] and was expressed per kilogram of estimated metabolic body size (FFM + 17.7 kg) [22].

#### 2.2.4. Adipose Tissue Biopsies

AT biopsies were collected with the Mercedes lipoaspirate techniques under sterile conditions and local anesthesia was administered. Biopsy specimen were taken from the scABD region, between one- and two-thirds of the distance from the iliac spine to the umbilicus and from the scFEM area, on the anterior aspect of the thigh, between one- to two-thirds of the distance from the superior iliac spine to the patella. The tissue was immediately flash frozen in liquid N_2_. The collection procedures were consistent for all biopsy collections.

#### 2.2.5. RNA Isolation

RNA from ~ 100 mg of AT was isolated by column purification (QIAGEN) and the yield was determined by spectrophotometry (NanoDrop Technologies). 200 ng from each RNA sample was reverse transcribed to cDNA using the High Capacity cDNA Reverse Transcription kit (Applied Biosystems). Relative quantification of mRNA expression was analyzed using ABI PRISM 7900 (Applied Biosystems). Custom TaqMan gene expression microfluidic cards (Life Technologies) for CT-1 (Hs00173498_m1) and Collagen, type1, alpha1 (Hs00164004_m1) were used for analysis. Samples were run in triplicate. Expression levels were normalized to cyclophilin B (Hs00168719_m1).

### 2.3. Statistical Analysis

Data are presented as mean ± SD or SEM, with an α level set at 0.05; statistical tests were two-tailed. Simple associations were examined using Spearman’s correlation. Changes in variables between baseline and post-overfeeding were analyzed by paired t-tests. Descriptive statistics for the women with obesity (Study 1) [18] and mean changes with overfeeding in the men (Study 2) [19] have been previously reported. A subset of subjects from each study who had adequate adipose samples were included in this analysis.

## 3. Results

Study 1: Twenty-two women with obesity (30 ± 6 years of age; BMI 32.4 ± 2.5 kg/m^2^) were examined and scABD and scFEM AT depots were assessed. The descriptive statistics are included in Table 1.

**Figure 1:**
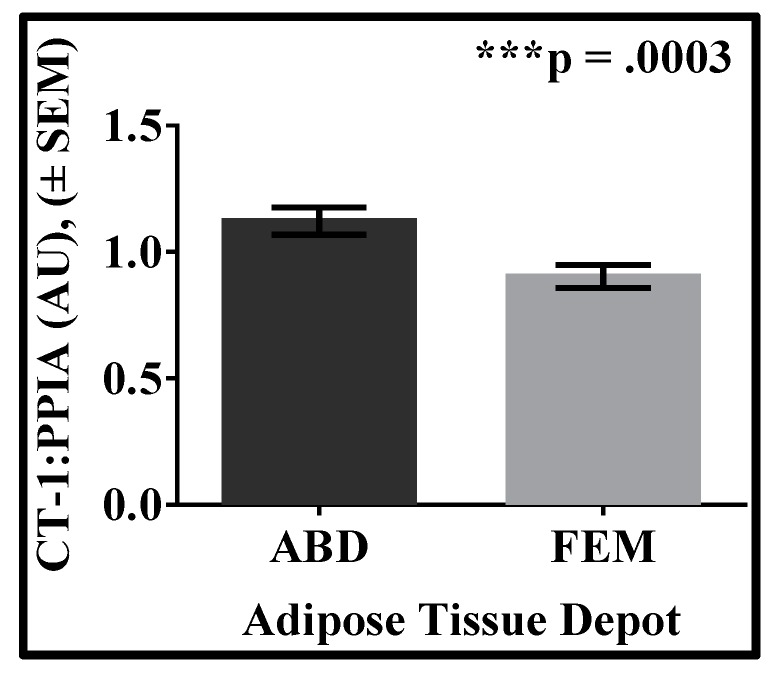
CT-1 expression is higher in the scABD than the scFEM AT depot in women with obesity. Subcutaneous abdominal (scABD) and femoral (scFEM) adipose tissue depots were collected from women with obesity (*N* = 22). RNA was extracted, and ~200 ng was processed for cardiotrophin (CT)-1 mRNA analysis. Samples were run in triplicate, and expression levels were normalized to cyclophilin B (PPIA) in arbitrary units (AU). Data are expressed as mean values ± SEM. The difference in CT-1 expression between scFEM and scABD depots is −0.28 (*p* = 0.0003).

**Figure 2:**
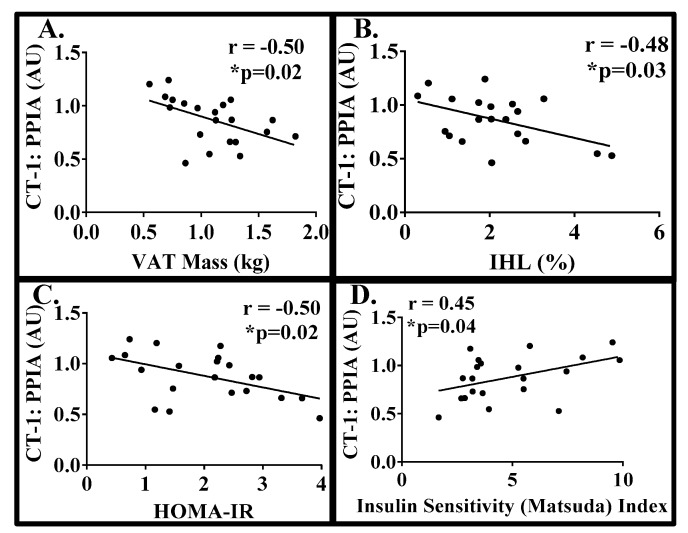
CT-1 scFEM expression is negatively associated with intra-abdominal adiposity, intrahepatic lipid, and HOMA-IR, and positively with Matsuda Index in women with obesity. Simple associations between scFEM CT-1 expression with ectopic fat and insulin sensitivity were analyzed using Pearson’s correlation (*N* = 22). (**A**) The Pearson’s correlation between scFEM CT-1 expression and VAT mass is −0.50 (*p* = 0.02). (**B**) The Pearson’s correlation between scFEM CT-1 expression and % IHL is −0.48 (*p* = 0.03). (**C**) The Pearson’s correlation between scFEM CT-1 expression and HOMA-IR is −0.50 (*p* = 0.02). (**D**) The Pearson’s correlation between scFEM CT-1 expression and Matsuda Index is 0.45 (*p* = 0.04).

We detected CT-1 mRNA levels in the subcutaneous AT of women with obesity, with higher expression in the scABD than the scFEM depot (Figure 1; *p* = 0.0003). Clinical data, including body composition, ectopic lipid and insulin sensitivity, from these women was used to examine potential correlations with CT-1 expression in both adipose depots. There were no significant correlations between BMI (*r* = 0.01; *p* = 0.93), % body fat (*r* = −0.09; *p* = 0.66), scABD AT Mass (*r* = −0.15; *p* = 0.48), VAT Mass (*r* = 0.01; *p* = 0.93), % IHL (*r* = 0.10; *p* = 0.63), HOMA-IR (*r* = −0.17; *p* = 0.40) or Matsuda Index (*r* = 0.31; *p* = 0.14) with scABD CT-1 expression. Conversely, Figure 2 demonstrates that scFEM CT-1 expression was negatively correlated with VAT mass (A; *r* = −0.50; *p* = 0.02), %IHL (B; *r* = −0.48; *p* = 0.03) and HOMA-IR (C; *r* = −0.50; *p* = 0.02), while positively associated with Matsuda Index (D; *r* = 0.45; *p* = 0.04). There were no associations with BMI (*r* = −0.26; *p* = 0.22), % body fat (*r* = −0.09; *p* = 0.66) or SAT Mass (*r* = −0.28; *p* = 0.21) with scFEM CT-1 levels. We did not observe any significant correlations of lipid cardiovascular risk factors, including TRIG (*r* = −0.17; *p* = 0.40), CHOL (*r* = −0.28; *p* = 0.19), HDL (*r* = −0.01; *p* = 0.91), LDL (*r* = −0.30; *p* = 0.16) or HDL:Total Chol (*r* = −0.22; *p* = 0.27), with scFEM CT-1 expression.

**Figure 3:**
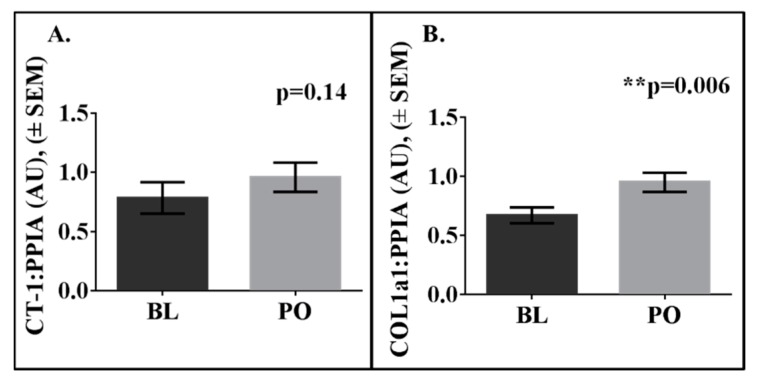
CT-1 is expressed in scABD AT in men, with no difference in expression between baseline and post-overfeeding. Subcutaneous abdominal (scABD) adipose tissue was collected from men (*N* = 15). RNA was extracted, and ~200 ng was processed for CT-1 and COL1a1 mRNA analysis. Samples were run in triplicate, and expression levels were normalized to cyclophilin B (PPIB). Data are expressed as mean values ± SEM. (**A**) The difference in CT-1 expression between post-overfeeding and baseline is 0.17 (*p* = 0.14). (**B**) The difference in COL1a1 expression between post-overfeeding and baseline is 0.28 (*p* = 0.006).

**Figure 4:**
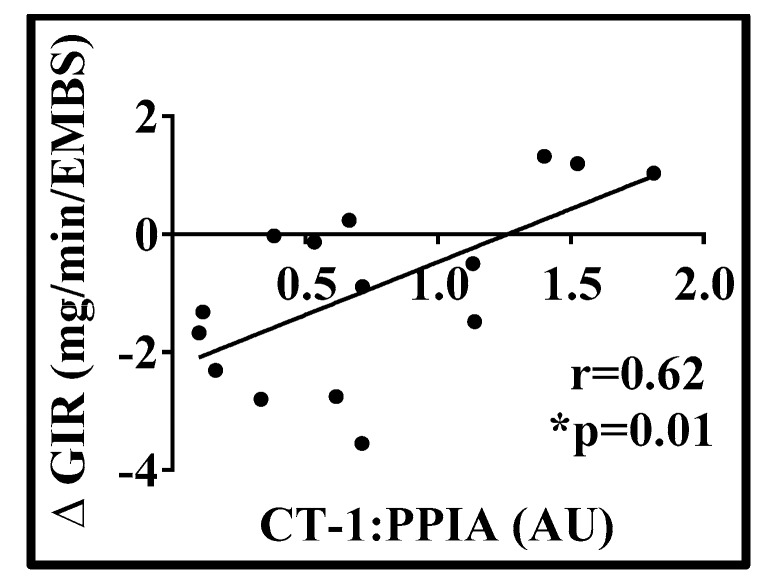
CT-1 scABD adipose expression is positively correlated with the change in GIR with 40% overfeeding in men. Simple associations between baseline scABD CT-1 expression and changes in insulin sensitivity in response to overfeeding were analyzed using Pearson’s correlation (*N* = 15). The hyperinsulinemic euglycemic clamp was used to assess glucose infusion rate (GIR) in the men. GIR with high-dose insulin infusion (50 mU/min/m^2^) was calculated and expressed as milligrams per minute per estimated metabolic body size (EMBS)). The Pearson’s correlation between CT-1 expression and the change in GIR is 0.62 (*p* = 0.01).

Study 2: Fifteen men that completed 8-weeks of 40% overfeeding (25 ± 4 years of age; BMI 24.7 ± 2.1 kg/m^2^) were examined and only the scABD was examined. Men gained an average of 7.73 ± 1.92 kg after 8-weeks of overfeeding. Mean changes with overfeeding are included in Table 2. As shown in Figure 3, we detected CT-1 mRNA levels in the scABD AT of men, with no statistically significant difference in expression at baseline versus post-overfeeding (4A; *p* = 0.14). Collagen 1 was included as a control, as expression levels of this extracellular matrix remodeling gene have previously been shown to increase in AT in response to overfeeding [23], as also demonstrated in the present analysis (4B; *p* = 0.006). Potential correlations were examined between scABD CT-1 expression at baseline and changes in clinical variables in response to overfeeding. There were no associations between baseline CT-1 expression with changes in % body fat (*r* = −0.08; *p* = 0.74), scABD AT mass (*r* = −0.24; *p* = 0.40), VAT mass (*r* = 0.10; *p* = 0.65) or IHL (*r* = 0.05; *p* = 0.84) (data not shown). Figure 4 demonstrates that CT-1 levels at baseline were positively correlated with the change in glucose disposal rate (insulin sensitivity) in response to overfeeding (*r* = 0.62; *p* = 0.01).

## 4. Discussion

A previous study that highlighted novel functions of CT-1 as a regulator of energy metabolism [12] prompted us to further characterize CT-1 expression in a cohort of men and women with available clinical outcome data. Distinct from other studies, our analyses examined AT CT-1 expression, as opposed to circulating levels. Novel findings in this study demonstrate for the first time that despite higher expression in the scABD, CT-1 expressed from the scFEM but not the scABD, depot was highly associated with features of metabolic health in women with obesity. Notably, higher adipose-derived CT-1 levels were associated with lower VAT mass and liver fat and greater insulin sensitivity. In addition, men with lower CT-1 adipose expression at baseline had a greater decline in insulin sensitivity in response to 8-weeks of 40% overfeeding. 

The relationship between CT-1 and obesity-related diseases remains unclear. Circulating levels of CT-1 were shown to be associated with hyperglycemia and elevated in individuals with impaired glucose tolerance and MetS [8,9]. Conversely, it has also been reported that CT-1 deficient mice had impaired metabolic features and administering CT-1 could reverse insulin resistance in obese mice, concluding that CT-1 had potential applications in the treatment of obesity and the MetS [12,24]. In humans, intense physical exercise has been shown to be directly associated with plasma CT-1 levels [25]. In the present analysis, we observed a negative association between CT-1 and ectopic fat (Figure 2) and a positive correlation with insulin sensitivity, as assessed by Matsuda Index, HOMA-IR and the hyperinsulinemic euglycemic clamp (Figure 2; Figure 4). Hence, our data strengthen previous findings that suggest that CT-1 may be related to improved metabolic health in humans. Although additional studies are necessary to establish whether CT-1 is a metabolically favorable cytokine, our present data provide new knowledge on the modulation of CT-1 in obesity, as well as during weight gain in response to a sustained hyper-caloric challenge. Interestingly, our data demonstrate that CT-1 expression is higher in the scABD than the scFEM depot. However, increased CT-1 expression from only the scFEM but not the scABD, depot was significantly correlated with improved metabolic health outcomes in women with obesity. Women display preferential adipose accumulation in the lower body (gluteal and scFEM) depots [15,16], which have been shown to be metabolically protective [17]. Hence, our findings suggest a potential role for CT-1 as a beneficial adipokine, whose expression from the favorable scFEM AT depot is highly correlated with relevant clinical metabolic outcomes in women with obesity.

CT-1 is known to be a secreted factor, however, various metabolic tissues, including AT, skeletal muscle and liver, are also targets of CT-1 action. CT-1 has been shown to promote insulin-stimulated glucose uptake in myotubes and cultured adipocytes [12], to modulate the production of other adipokines [26] and to exert protective effects against liver apoptosis [27], hepatocyte injury [28], hepatic steatosis [13] and renal damage [29]. These studies provide further evidence to support the beneficial effects of CT-1.

In relation to cardiovascular disease, studies have proposed that plasma levels of CT-1 could be applied in the diagnosis of hypertensive heart disease [30] and may be a predictor of mortality in patients with chronic heart failure [31]. Interestingly, we did not observe any significant correlations of lipid cardiovascular risk factors (see Results section).

We present the first analysis to examine CT-1 expression in distinct adipose depots of humans, which represents a significant strength of our study. Notably, we revealed striking depot differences in both CT-1 expression and its relationship with clinical outcomes in women with obesity. We did not have scFEM adipose available from men; therefore, depot differences in this cohort could not be assessed. The extensive phenotyping of men and women is an additional strength, as we could explore novel relationships between CT-1 and a variety of important health outcomes. There are also several limitations to our study, including a small sample size. However, our subject population includes both men and women with clinically significant characteristics who are either obese or in positive energy balance resulting in substantial weight gain. Another limitation is that we only assessed mRNA expression and due to limited tissue availability, did not measure protein expression. Finally, it is important to note that our results reflect correlations and do not establish causation. Nevertheless, our novel observations support previously reported data and suggest that CT-1 may play a physiological role in obesity and metabolic disorders in humans.

## 5. Conclusions

In conclusion, findings from this study reveal that CT-1 expressed from the scFEM, not the scABD, AT depot was negatively associated with ectopic lipid (visceral adiposity and liver fat) and positively correlated with insulin sensitivity in women with obesity. Furthermore, men with higher CT-1 levels in the adipose at baseline had less of a decline in insulin sensitivity in response to 8-weeks of a hyper-caloric challenge. Hence, these observations implicate CT-1 as a potential mediator of energy metabolism in humans. Of note, data from a randomized clinical trial (NCT01334697) evaluating the safety, tolerability and pharmacokinetics of the administration of recombinant human CT-1 versus placebo in healthy volunteers may offer needed insight to the metabolic functions of CT-1. Promising results in humans from this study and others might confer CT-1 as a potential therapeutic.

## Figures and Tables

**Table 1:** Descriptive Statistics for Study 1.

Variable	Mean	SD
Age	30	6
Body weight (kg)	87.16	8.17
BMI (kg/m^2^)	32.40	2.48
Body Fat (%)	45.19	4.29
scABD AT (kg)	7.84	1.60
VAT (kg)	1.09	0.33
IHL (%)	2.13	1.18
Matsuda Index	4.66	2.44
HOMA-IR	2.04	1.00
Mean BP (mmHg)	76.19	6.74
TRIG (mg/dL)	82.45	41.44
Total CHOL (mg/dL)	180.13	41.79
LDL (mg/dL)	109.9	32.71
HDL (mg/dL)	53.74	13.82
Total CHOL:HDL	3.47	0.93

**Table 2:** Clinical Changes with Overfeeding (*N* = 15).

VARIABLE	BASELINE	POST-OVERFEEDING	Change	*p* value
	Mean ± SD	Mean ± SD		
Body Weight (kg)	79.71 ± 7.33	87.44 ± 7.83	7.73 ± 1.92	<0.0001
BMI (kg/m^2^)	24.72 ± 2.09	27.37 ± 2.24	2.65 ± 0.91	<0.0001
Body Fat (%)	21.20 ± 4.28	24.55 ± 4.57	3.35 ± 0.96	<0.0001
scABD AT (kg)	4.38 ± 1.34	5.88 ± 1.67	1.50 ± 0.50	<0.0001
VAT (kg)	0.54 ± 0.34	0.93 ± 0.45	0.39 ± 0.16	<0.0001
IHL (%)	0.69 ± 0.41	1.27 ± 1.11	0.58 ± 0.88	0.02
Glucose Infusion (mg/min∙(FFM+17.7))				
10 (mU/min·m^2^ insulin)	2.62 ± 0.87	2.08 ± 0.67	−0.54 ± 0.53	0.001
50 (mU/min·m^2^ insulin)	11.61 ± 2.83	10.70 ± 2.82	−0.90 ± 1.53	0.03

## Abbreviations

Adipose Tissue, AT; Body Mass Index, BMI; Blood Pressure, BP; Cholesterol, CHOL; High- Density Lipoprotein, HDL; Intrahepatic Lipid, IHL; Low-Density Lipoprotein, LDL; Subcutaneous Abdominal Adipose Tissue, scABD AT; Visceral Adipose Tissue, VAT; Triglycerides, TG.
